# Analysis of Guangzhou city image perception based on weibo text data (2019–2023)

**DOI:** 10.1016/j.heliyon.2024.e36577

**Published:** 2024-08-21

**Authors:** Huimin Qu, Bor Tsong Teh, Nikmatul Adha Nordin, Zhuqin Liang

**Affiliations:** a Centre for Sustainable Urban Planning and Real Estate (SUPRE), Faculty of Built Environment, Universiti Malaya, 50603, Kuala Lumpur, Malaysia; b School of Mathematical Sciences, Universiti Sains Malaysia, 11800, Penang, Malaysia

**Keywords:** City image, Social media, Text mining, Natural language processing

## Abstract

With the popularization of smart mobile terminals and social media, a large amount of data containing textual information about the city has been generated on social media platforms, covering all areas of the city. This provides a new way for the study of comprehensive perception of city image. In the Internet era, users express their opinions about cities through social media platforms (e.g., Sina Weibo), and mining this information helps to understand the image of cities on mainstream social media and to target positive images to improve the competitiveness of the city's image. In this paper, 370,000 microblog messages related to “Guangzhou City” between 2019 and 2023 are collected using web crawler technology, and three typical text analysis methods are adopted: Term Frequency-Inverse Document Frequency (TF-IDF), Latent Dirichlet Allocation (LDA), and Sentiment Analysis (SnowNLP), to understand the characteristics of Guangzhou city image. gain an in-depth understanding of Guangzhou's urban image characteristics. The study shows that extensive data analysis methods based on text mining can perceive the dynamics and trends of the city in a timely manner, refine the characteristics of Guangzhou's urban image, and propose communication strategies for Guangzhou's image. This study aims to mine Guangzhou's urban image presented on Weibo, provide data support for relevant departments in China and Guangzhou to formulate communication strategies, and provide references for other cities to manage their urban image.

## 1 Introduction

The concept of "image" encompasses people's abstract feelings and perceptions about a particular region or place, forming a psychological representation that includes impressions, opinions, cognition, evaluation, and feelings. In the 1960s, Lynch introduced a method for studying urban images by employing visual perception theory, conducting surveys, and creating cognitive maps. This approach identified five key elements shaping the urban image [1]. Previous research heavily relied on time-consuming field investigations with limited sample sizes. Two predominant methodologies emerged: one is to perceive the spatial image of the city concerning Lynch's cognitive map and other methods and to re-study the five elements [[2], [3], [4], [5], [6]]; The other is to obtain the perception of different image attributes using mathematical statistics analysis and qualitative analysis [[7], [8], [9], [10], [11], [12]].

The Internet era revolutionised the study of urban images, bringing new data and methods [13,14]. With their originality, timeliness, simplicity, and openness, online platforms like Weibo have become crucial for information exchange and social relations. While beneficial, Weibo's vast data source presents challenges for manual analysis, and it is necessary to develop natural language processing technology, which is why the development of natural language processing technology has become crucial. New tools enable large-scale data analysis, thus providing technical support for urban imagery research. By excavating the content of Weibo, we can gain a better understanding of a city's current image, which can provide the basis for governmental departments to make decisions and improve the city's image in a targeted way [15].

In South China, Guangzhou is a prosperous metropolis of significant economic, cultural, and historical significance. The history of Guangzhou can be traced back to the period of Nanyue State more than 2000 years ago, and now Guangzhou has developed into a modern international city. Located in the hinterland of the Pearl River Delta, Guangzhou is an important transportation hub and international trade gateway [16]. The city has led the modernisation process in the southern region, witnessed substantial economic growth, and cultivated a diversified industrial landscape, including manufacturing, service and information technology. Culturally, Guangzhou is full of vitality, seamlessly blending traditional and modern elements. Its unique Lingnan culture is evident in its architecture, food, and language. As a famous tourist destination, Guangzhou attracts people's attention with its historical landmarks and modern scenic spots [17].

In this paper, Guangzhou is taken as the investigation object, and the tweets related to "Guangzhou" from 2019 to 2023 are obtained by using web crawler technology. These tweets have gone through pre-processing steps such as language recognition, language transcription and word separation. In the study, we use a combination of TF-IDF, LDA and Sentiment Analysis, aiming to explore the city image of Guangzhou City in depth. First, we used Octopus software to capture the relevant texts about Guangzhou city posted by users on Weibo. Through data preprocessing, TF-IDF feature extraction and weight calculation were performed to quantify the importance of words in the text data. This step helps to gain insight into the contribution of keywords in tweets to the city image. Second, we built an LDA topic model to identify potential topic structures in the text data. Through this step, we expect to reveal the hidden themes in the tweets, thus providing a more comprehensive understanding of the multidimensional face of Guangzhou City. Further, we used sentiment analysis to explore the correlation between feature words and users' emotional orientation under each perception dimension. This analysis helps to understand the public's affective tendencies towards the city of Guangzhou and provides useful references for sustainable development and innovation and communication paths for the city in the future.

This paper's main contributions are as follows. (1) This study utilizes advanced web crawler technology to systematically collect a large amount of tweets related to “Guangzhou City” on Weibo between 2019 and 2023. This method ensures the comprehensiveness and real-time nature of the dataset, which truly reflects the public's views and opinions on Guangzhou. (2) By integrating TF-IDF, LDA and Sentiment Analysis, this study provides a multi-dimensional analysis methodology: TF-IDF quantifies the importance of keywords in the tweets, LDA identifies the potential themes, and Sentiment Analysis reveals the emotional tendencies associated with these themes. (3) The combination of these analytic techniques provided a detailed and in-depth understanding of Guangzhou's urban image. The study not only reveals the major themes and keywords associated with Guangzhou, but also the sentiment tendencies behind these themes, which helps to provide a comprehensive understanding of how the public feels about Guangzhou. (4) The findings provide valuable insights for policy makers and urban planners. By identifying key areas of public concern and emotional tendencies towards different aspects of the city, this study provides actionable data that can guide targeted improvements and strategic initiatives to enhance Guangzhou's urban image and sustainable development. (5) This study advances urban image research by demonstrating the potential of modern digital tools and social media data. This study emphasizes the effectiveness of using online platforms and natural language processing techniques for large-scale, real-time city image analysis, providing new research perspectives for other cities and regions. (6) The methodology and findings of this study laid the foundation for future research on city image analysis. The integration of web crawlers, advanced text analysis techniques and sentiment analysis serves as a powerful framework that can be advanced and improved in different urban contexts. (7) There is a small body of research literature on Guangzhou's urban image, and this paper further expands the research in this field.

The structure of this paper is as follows. The second part sorts out the related literature in this field. The third part introduces the basic theories and research methods involved in this study. In the fourth part, more than 370,000 tweets related to "Guangzhou City" were collected by web crawler, and the collected texts were quantitatively analyzed by using natural language processing technology. The fifth part is the result of further discussion, which analyzes and summarizes the current urban characteristics of Guangzhou's urban image, and puts forward the strategies to improve the urban image. The sixth part summarizes the main findings, points out the significance and limitations of this study, and looks forward to future research directions.

## 2 Literature review

### 2.1 Social media and urban image perception

Social media has become a pivotal resource for studying user behaviour, social trends, and various phenomena related to urban image perception [18]. Researchers employ diverse methods utilizing social media data. For instance, Cranshaw et al. focus on understanding urban dynamics through social media data analysis [7], while Hollenstein and Purves use tag data and geographic information from online photos to identify urban centers and demarcate boundaries [19]. Hasan et al. analyze Twitter's geographic tag data to unveil urban residents' geographical perception dynamics [20], and Silva et al. utilize Flickr's geographic tag photos to study people's perceptions of urban scenes [21]. Quercia et al. construct a city image vocabulary based on commonly used words and sentences when describing cities [22], and Crandall et al. explore urban space perception in different communities using Flickr's photos with geographical labels [23]. Studies also delve into public perceptions of urban style by analysing geographical label photos on Instagram [24]. Hochman & Manovich analyze Tel Aviv's Instagram pictures to reveal the city's social, cultural, and spatial characteristics [25]. Stefanidis et al. estimate urban population mobility and traffic patterns using geo-location data from Twitter [26], while Hasan et al. discuss urban human activities and mobile patterns with large-scale geographic positioning data from social media [20]. Liu et al. contribute to revealing urban functional zoning with social media data [27]. Collectively, these studies underscore that social media big data offers an effective means to comprehensively understand public cognition and perception of urban images, providing valuable insights for urban planning and design in the contemporary data-driven environment. Thus, understanding the current city image of Guangzhou through mainstream social media is one of the key links to enhance the overall image of Guangzhou.

### 2.2 Text mining and urban image analysis

Text mining plays an increasingly important role in the study of city image. Researchers can deeply understand the public's views, emotions and concerns about the city by analysing text data such as social media, news reports, blogs and comments. Weibo text data has played a vital role in public opinion generation and evolution mechanism, public opinion monitoring and prediction, and emotional perception, and has also made remarkable achievements in the study of urban issues. Taking Weibo as an example, the researchers found a correlation between the city's image and the spread of the second crisis. By studying the evolution mechanism of the second crisis in Weibo, Luo & Zhai found that the theme of Weibo changed from a political event to a more negative tourism boycott, which triggered an essential impact on urban tourism and even involved the conflict between China, Hong Kong and mainland people [28]. In emotional analysis, by analysing the tweets of city-related keywords, the researcher reveals the public's emotional tendency towards the city. Taking New York as an example, Smith and others conducted emotional analysis through big data on Twitter and deeply explored citizens' attitudes and feelings towards the city. This analysis reflects the emotional experience of urban residents and captures the subtle differences in urban image changes in different events, seasons or periods [29]. In urban planning, researchers can also understand the use and activity of urban space by mining the geographic information of tweets. Johnson et al. used the geographic tag information of tweets to draw the spatial distribution map of urban hot spots, which provided valuable insights for urban planners about public meeting places and cultural activity centers [30]. In addition, the research of Lansley & Longley found the relationship between Twitter theme and geographical location through the theme modelling of Twitter text, which provided a new perspective for the study of urban functional characteristics [31]. Therefore, this study examines the city characteristics from the perspective of Guangzhou's urban image and proposes image shaping countermeasures to enhance the city's urban competitiveness.

## 3 Model methodology

### 3.1 Data preprocessing and word segmentation

To ensure data quality and algorithm compatibility, rigorous data cleaning and word segmentation are essential before delving into text analysis. English text undergoes word separation using the "word_tokenize()" function from the Natural Language Toolkit (NLTK) in Python. Enhancements include preprocessing for URLs, emoticons, special symbols, "@", "#", word form reduction, synonym replacement, phrase recognition, and deactivation. Chinese information is extracted using regular expressions, and the Jieba word segmentation system accurately segments Chinese-extracted Weibo text. The term "Guangzhou City" is included in the list of stopped words to prevent bias.

### 3.2 Term Frequency-Inverse Document Frequency (TF-IDF)

TF-IDF algorithm is a classic algorithm in text keyword extraction, which is widely used in text retrieval and text mining and is an effective weighting technology. The algorithm evaluates the importance of a word or word in the whole text or corpus by counting the number of times it appears in the text or expected set [32]. Among them, Term Frequency (TF) indicates the frequency of a given word appearing in the text. Specifically, the number of times the word appears in the text is divided by the total number of words. To prevent articles with extended word frequency, we can normalise them. This normalisation can be achieved by dividing the word frequency by the total number of all words in the text [33]. Expressed explicitly by Formula (1):

(1) $$tf_{i}=\frac{n_{i,j}}{\sum_{k}n_{k,j}}$$

$tf_{i}$ represents the ratio of the number of occurrences of a given word in a given file to the number of occurrences of all words in this file. Among them, the variable $n_{i,j}$ represents the number of occurrences of the word $t_{i}$ in the article $d_{j}$, and the denominator represents the sum of the number of occurrences of all words in the document $d_{j}$. Meanwhile, the Inverse Document Frequency (IDF) is used to measure the importance of a word in the whole corpus. The greater the value, the less the number of texts containing the featured item, and it also shows that the entry has strong representativeness and discrimination. The calculation formula (2) of the IDF value is as follows:

(2) $${\;idf}_{i}=\log\frac{\left|D\right|}{|\{j:t_{i}\epsilon d_{j}\}|}$$

Where |D| represents the total number of files in the corpus, $|\{j\text{:}t_{i}\epsilon d_{j}\}|$ represents the number of files containing the word $t_{i}$, and ${idf}_{i}$ calculates the ratio of the total number of files in the corpus to the number of files containing a given word.

Then, the TF-IDF formula (3) is expressed as:

(3) $$tf_{i,j}idf_{i}=tf_{i,j}\times idf_{i}$$

Therefore, the TF-IDF with a high weight should meet the two conditions of the high frequency of words appearing in the full text (high TF value) and a small number of files containing the words (high IDF value).

### 3.3 Latent Dirichlet Allocation topic model (LDA)

LDA topic model is a text representation model considering semantic relevance. The topic extraction effect of LDA topic model is directly affected by the number of potential topics, and two commonly used evaluation methods to determine the optimal number of topics are based on coherence and perplexity [34]. The coherence score measures the quality of the topic model by calculating the consistency of document topic distribution. Among them, a popular coherence measure is the UCI (Umass Coherence Index) coherence [35]. The calculation formula (4) is as follows:

(4) $$UCI\;Coherence=\frac{2}{N\times \left(N-1\right)}\sum_{i=1}^{N-1}\sum_{j=i+1}^{N}\;\log\frac{P\left(w_{i},w_{j}\right)+\in }{P\left(w_{i}\right)P\left(w_{j}\right)}$$

Where N is the number of topics, $w_{i}$ and $w_{j}$ are two different words, and $P\left(w_{i},w_{j}\right)$ is the joint probability of the words $w_{i}$ and $w_{j}$ under the same topic. $P\left(w_{i}\right)$ and $P\left(w_{j}\right)$ are marginal probability of the words $w_{i}$ and $w_{j}$, respectively, and ∈ is a smooth term, which is usually set as a small positive number to avoid the error of dividing by zero in logarithm. This formula measures the probability of word pairs appearing together under the same topic relative to their probability of appearing alone in the corpus.

Perplexity is an important index used to evaluate the performance of the LDA model, which is often used to measure the model's ability to predict new data. The lower the value, the more accurate the model is in predicting new data, which reflects the model's uncertainty to the article's topic [[36], [37], [38]]. Its calculation formula (5) shows.

(5) $$Perplexity=\exp\left(-\frac{1}{N}\sum_{i=1}^{N}\log\;P\left(w_{i}\right)\right)\;$$

Where N represents the total number of words in the document, and $P\left(w_{i}\right)$ represents the prediction probability of the model for the i word. The lower the degree of confusion, the more accurately and reliably the model predicts new data. In the model construction process, this paper chooses topics with greater coherence and less confusion to complete the construction of the LDA theme model. This choice is based on the comprehensive consideration of coherence and confusion, which helps improve the model's performance and the prediction accuracy of new data.

### 3.4 SnowNLP sentiment analysis

Sentiment analysis extracts attitude tendencies from texts, and SnowNLP, a Python-based tool, is employed for sentiment analysis in Chinese text processing. Standard methods include the simple Bayesian approach, Long Short-Term Memory (LSTM), and Valence Aware Dictionary and Sentiment Reasoner (VADER) [[39], [40], [41]]. SnowNLP offers features like word segmentation, part-of-speech tagging, and sentiment analysis, utilizing statistical models trained on diverse Chinese corpora. Its sentiment analysis employs a naive Bayesian classifier, learning from labeled training data to predict emotional tendencies and generate emotional scores [41,42]. The analysis considers emotional words, measuring intensity through punctuation, degree adverbs, negation, and conjunctions.

## 4 Empirical research

### 4.1 Data collection

This study utilizes the API developed by Sina Weibo (https://weibo.com/home, accessed on January 2, 2024) as the primary data source. Through Python programming, we extracted tweet links containing the keyword "Guangzhou City" from January 1, 2019, to December 31, 2023. The text content was then crawled using Octopus software. Over the specified period, a total of 377,430 tweets focused on the theme of "Guangzhou City" were collected, encompassing Weibo text, release dates, user information, and Weibo ID. Table 1 illustrates the distribution of tweets per year.

**Table 1:** Statistics on the number of tweets from 2019 to 2023.

Year	Number of Sina Weibo
2019	80,957
2020	73,509
2021	71,620
2022	60,365
2023	90,979
Total	377,430

### 4.2 Data collection

Python's NLTK module handles word segmentation in data processing, while the collections module is employed for word frequency statistics. The elimination of non-significant words is based on statistical results, involving removing special characters, word segmentation, and stop word removal. Common terms such as "Guangzhou" and "city" are excluded from word frequency analysis due to their pervasive presence in tweets. Following preprocessing, a second round of word segmentation occurs. Subsequently, TF-IDF keyword statistics and the LDA theme model are employed for theme mining and sentiment analysis.

### 4.3 Keywords statistics

Table 2 shows the top 50 keywords in 2019. The list of characteristic words includes words related to cities and countries, such as 'Shanghai', 'China' and 'Beijing', and words with high TF-IDF values, ' The Greater Bay Area' and 'house price', which reflects that users in Weibo paid close attention to urban development, scientific and technological progress and economic situation in 2019. Among the characteristic words with the highest total TF-IDF value, cities such as 'Guangzhou', 'Beijing' and 'Shenzhen' have attracted the attention of users on Weibo, and 'Weibo' itself, as a characteristic word highlights the core position of social media in user communication and information dissemination. These TF-IDF data provide powerful clues for understanding the hot topics that Weibo users pay attention to in 2019.

**Table 2:** Top 50 keywords of 2019.

NO	Keywords	TF-IDF Value	Occurrences	NO.	Keywords	TF-IDF Value	Occurrences
1	Shanghai	6775.33215339	28330	26	show	1755.21909716	7156
2	Beijing	6605.74392634	27890	27	center	2303.37631541	6804
3	Shenzhen	7010.53651871	26945	28	economy	2047.38552839	6359
4	China	6205.98015691	17957	29	Zhengzhou	2180.55879849	6285
5	Hangzhou	4098.20096462	15997	30	build	2160.27443061	6093
6	2019	4562.30844433	15948	31	report	1959.17034852	6081
7	Chengdu	4436.99522552	15511	32	exceed	1796.01007547	5975
8	whole country	4162.08993257	13310	33	Guangdong	2777.17038133	5855
9	Chongqing	3247.18933217	12069	34	market	1932.56719216	5497
10	Wuhan	3338.8141902	11847	35	ranking	1950.63999651	5216
11	Nanjing	3003.92283009	11313	36	international	1847.28621294	5197
12	issue	2566.00064638	10601	37	service	1917.88762657	5116
13	develop	3352.26218465	10199	38	travel	2466.27357041	5061
14	Xi'an	2995.06454973	9584	39	country	1659.54097628	4992
15	video	4321.50905398	9434	40	Hong Kong	1997.67142242	4618
16	life	4566.53182278	9075	41	enterprise	1587.17986107	4521
17	Tianjin	2379.35073231	8552	42	activity	2014.59048715	4505
18	interlinkage	3363.02708457	8465	43	culture	1820.77481749	4503
19	first-tier	3250.87639871	8449	44	project	1713.36487754	4136
20	webpage	3149.05704517	8208	45	subway	2451.06922674	3822
21	work	3244.455661	7700	46	company	1579.40880734	3784
22	Weibo	3438.25146371	7689	47	The Greater Bay Area	1656.99772752	3381
23	time	3027.89673993	7325	48	house price	1630.87736421	3013
24	data	2006.96520697	7229	49	rise	1409.08766359	2641
25	2018	2287.48166875	7217	50	5G	1192.22003552	1579

Table 3 shows the top 50 keywords in 2020. Regarding TF-IDF value, words such as '2020′, 'epidemic',' first-class' and 'China' have relatively high values, which shows that the importance of these words in Weibo lies not only in frequency but also in their uniqueness. This is in line with the phenomenon that '2020′ is a particular period, and the epidemic situation has caused the attention of first-tier cities to increase, highlighting the global influence of COVID-19. Other characteristic words with relatively high TF-IDF values, such as 'college', 'airport', 'market' and 'service', reveal Weibo users' concerns in academic, transportation and market fields.

**Table 3:** Top 50 keywords of 2020.

NO	Keywords	TF-IDF Value	Occurrences	NO.	Keywords	TF-IDF Value	Occurrences
1	Shanghai	5718.67751403	23993	26	interlinkage	2553.97834646	6530
2	Shenzhen	6555.96653878	23669	27	Tianjin	1787.14470489	6195
3	Beijing	5531.83361247	22580	28	webpage	2349.82559878	6154
4	China	5315.75289599	14686	29	center	2212.20521159	6139
5	Hangzhou	3828.84260059	14623	30	Guangdong	2829.73084417	6034
6	whole country	4685.78768296	14514	31	Zhengzhou	1831.88870882	5998
7	Chengdu	3803.781054	14237	32	2019	1798.83668451	5858
8	2020	3849.83793339	12330	33	first	1708.18931359	5854
9	video	4975.49887361	12018	34	exceed	1677.14062128	5506
10	Chongqing	3200.55450326	11477	35	situation	1544.31489541	5473
11	Wuhan	3638.82781725	11351	36	market	1907.37261086	5367
12	Weibo	4317.6312915	10957	37	many	2417.16063209	5363
13	Nanjing	2677.66687162	9869	38	service	1968.52836386	5130
14	develop	3246.81019636	9124	39	build	2057.46443601	5088
15	issue	2448.95032086	8936	40	country	1590.97756825	4860
16	epidemic	3439.44175072	8560	41	report	1617.06009057	4811
17	Xi'an	2523.00477507	8456	42	international	1646.03060567	4464
18	work	3206.70644085	8124	43	enterprise	1668.05276728	4460
19	first-tier	3025.91563434	7846	44	project	1858.63299283	4038
20	life	3648.50910486	7823	45	company	1565.06386462	3759
21	Changsha	2292.88740693	7438	46	house price	1671.53206624	3276
22	data	2001.19977199	7366	47	rise	1498.73890987	3063
23	economy	2269.74033531	7087	48	airport	1500.89001851	2755
24	time	2583.96613634	6802	49	university	898.74680432	1713
25	show	1643.19420611	6678	50	college	512.77884729	1027

Table 4 shows the analysis of Weibo's TF-IDF data in 2021. Among the top 50 keywords, words such as 'Shanghai', 'China', 'Beijing', 'Guangdong', 'house price' and 'epidemic' have become the main focus of attention. The three words 'Wuhan', '2020′, and 'Epidemic', with their relatively high TF-IDF values, reflect their uniqueness and attention in Weibo's topic. This is consistent with the phenomenon that 2020 is a particular period, and the global attention caused by the epidemic has increased. In addition, the data in 2021 also showed some new concerns, such as 'doctor', 'hospital', 'oral', 'orthodontics', 'tooth' and other words, which showed Weibo users' concerns about medical care, oral health and other fields. Words like 'enterprise', 'market' and' economy' reflect users' concerns about business and economic trends.

**Table 4:** Top 50 keywords of 2021.

NO	Keywords	TF-IDF Value	Occurrences	NO.	Keywords	TF-IDF Value	Occurrences
1	Shenzhen	6506.1567365	26623	26	center	2335.90866242	6296
2	Shanghai	5722.17347047	25350	27	economy	1917.22024047	6013
3	Beijing	5603.18549036	24507	28	exceed	1785.96763858	5911
4	Chengdu	4083.77773301	16217	29	epidemic	2818.96583992	5738
5	Hangzhou	3704.82711037	15602	30	build	2175.30133242	5724
6	whole country	4094.35235263	13470	31	Dongguan	1950.19587741	5457
7	video	4862.91025563	13026	32	hospital	1238.66687758	5435
8	China	4653.55411427	12468	33	Guangdong	2466.66336212	5367
9	Weibo	4104.99503476	12440	34	market	1899.81125827	5089
10	2021	3423.08277264	11155	35	country	1523.83873234	4774
11	develop	3450.29735606	9874	36	region	1526.45775292	4642
12	Chongqing	2900.91121617	9206	37	Foshan	1866.78184101	4625
13	Nanjing	2612.0367293	8768	38	international	1670.20310506	4554
14	Wuhan	2525.7384224	8323	39	doctor	1052.71702393	4393
15	data	2306.17509953	8285	40	project	1912.2901362	4311
16	issue	2381.45446198	8167	41	enterprise	1501.49641727	4158
17	work	3111.50338193	7891	42	correct	2168.25881145	4122
18	first-tier	3036.53148316	7753	43	tooth	2463.69387488	4026
19	life	3576.33980442	7127	44	house price	1855.52639453	3901
20	Xi'an	2310.95754091	7125	45	subway	2280.25099398	3707
21	Zhengzhou	1675.6185229	7084	46	orthodontics	662.96319842	3661
22	2020	2157.05257645	6747	47	Which	689.34562726	3510
23	time	2366.48661797	6657	48	company	1440.29534021	3451
24	interlinkage	2275.56056804	6568	49	rise	1674.05641638	3288
25	service	1925.93191268	6487	50	oral cavity	643.9477278	2933

Table 5 shows the analysis of TF-IDF data in Weibo in 2022. Among the top 50 characteristic words, words such as 'Shanghai', 'China', 'Beijing', 'Guangdong' and 'Epidemic' have attracted significant attention. Regarding the epidemic, words such as 'nucleic acid', 'detection' and 'prevention and control' also show relatively high TF-IDF values, reflecting persistent epidemic concern. In the economic field, words such as 'enterprise', 'market' and 'construction' show users' concern about economic development and urban construction. In addition, users have also been widely concerned with words such as 'life', 'housekeeping' and 'logistics', revealing Weibo's concern about diversified life as an information platform.

**Table 5:** Top 50 keywords of 2022.

NO	Keywords	TF-IDF Value	Occurrences	NO	Keywords	TF-IDF Value	Occurrences
1	Shenzhen	4818.99696142	17132	26	Guangdong	2255.36007865	4931
2	Shanghai	4493.63383524	16956	27	data	1583.30810653	4895
3	Beijing	4084.54501847	16166	28	provide	1120.79318805	4815
4	Chengdu	2916.34251881	10862	29	region	1493.52121315	4649
5	whole country	3116.63795094	10688	30	Dongguan	1319.86839066	4558
6	Weibo	3847.65087018	10322	31	zone	1013.93929453	4496
7	2022	2883.01030072	10062	32	centre	1485.52286735	4433
8	video	4159.60794494	10042	33	hour	1526.75825235	4387
9	China	3707.93930957	10004	34	country	1348.08181337	4323
10	Hangzhou	2628.46667715	9821	35	interlinkage	1575.04039048	4213
11	Chongqing	2391.69312169	8585	36	build	1653.12563237	4132
12	epidemic	3586.28361151	7978	37	market	1410.88607112	4026
13	life	3325.2950699	7873	38	webpage	1438.54424004	3892
14	Wuhan	2039.86710276	7718	39	health	905.23792	3714
15	work	2396.76527108	7506	40	project	1685.6467738	3618
16	Nanjing	1978.87097209	7360	41	personnel	1134.44962162	3605
17	develop	2516.13886145	7288	42	enterprise	1192.5704375	3599
18	Xi'an	1771.37443351	6820	43	company	1258.86663834	3492
19	issue	1813.46072015	6642	44	prevention and control	1310.90995431	3439
20	service	2233.27025685	6186	45	nucleic acid	1537.12266879	3348
21	time	2057.33610798	6060	46	product	1232.85117088	3267
22	Changsha	1682.55803007	5888	47	subway	1788.15912179	3069
23	first-tier	2449.48147702	5391	48	examine	860.77877981	2416
24	economy	1553.96347159	5194	49	logistics	478.31803169	1034
25	2021	1708.99582777	5026	50	household management	293.30978958	316

Table 6 presents the TF-IDF data analysis on Weibo in 2023. Keywords like '2023,' 'first-tier,' 'Shanghai,' 'China,' 'Beijing,' 'Guangdong,' 'Chengdu,' and 'Shenzhen' exhibit high TF-IDF values, indicating users' keen interest in these areas and their concern for first-tier cities and national development. Additionally, '100 million yuan' and 'policy' with high TF-IDF values suggest users' sensitivity to financial scale and policy changes, reflecting concerns about financial and political matters. 'Study abroad' and 'culture' also contribute significantly to the total TF-IDF value, possibly indicating users' interest in international education and cultural exchange. Notably, housekeeping and daily life keywords reflect users' concerns about practical matters and family services.

**Table 6:** Top 50 keywords of 2023.

NO	Keywords	TF-IDF Value	Occurrences	NO	Keywords	TF-IDF Value	Occurrences
1	Shanghai	5978.95093494	24080	26	select	1993.74257071	6506
2	Shenzhen	6446.0731945	23506	27	many	2641.00763417	6397
3	Beijing	5732.99273999	22247	28	market	1875.26412397	6034
4	Weibo	4793.49776995	17263	29	travel	2567.43695161	6024
5	video	5901.75488782	17213	30	2022	1915.71787802	5966
6	Chengdu	4036.80107204	14717	31	service	1739.64134841	5933
7	2023	5735.15874735	14284	32	economy	1576.74271923	5803
8	whole country	3849.82958349	13856	33	grow	1717.06855775	5579
9	China	4527.72362957	13678	34	project	1989.79130802	5477
10	Hangzhou	3863.77846977	13588	35	centre	1713.87372781	5402
11	Chongqing	2722.15434297	10078	36	The Greater Bay Area	1876.06597293	5276
12	Wuhan	2697.52488278	9383	37	country	1264.08182204	5242
13	Nanjing	2461.89078976	9217	38	build	1847.80199676	5239
14	develop	2986.3348031	9104	39	activity	2168.99763395	5077
15	data	2109.2722118	8420	40	enterprise	1588.4251637	4967
16	first-tier	3329.50911763	8390	41	policy	1982.79522221	4602
17	show	1946.23400322	8172	42	culture	1860.75031933	4459
18	Changsha	2476.06591187	8086	43	subway	2705.11578382	4127
19	Xi'an	2176.07756563	8054	44	Year-on-year	1372.08499971	4061
20	issue	2068.53217618	7963	45	Hong Kong	1644.80702989	3836
21	time	2931.71758387	7961	46	consume	1341.22290747	3741
22	Guangdong	3475.09457171	7848	47	company	1553.6421165	3659
23	life	3455.69660802	7566	48	hundred million yuan	1088.62482932	2912
24	work	2507.84857678	6891	49	study abroad	1251.48944887	1465
25	real	3942.55456752	6507	50	household management	572.3218987	603

**Fig. 1:**
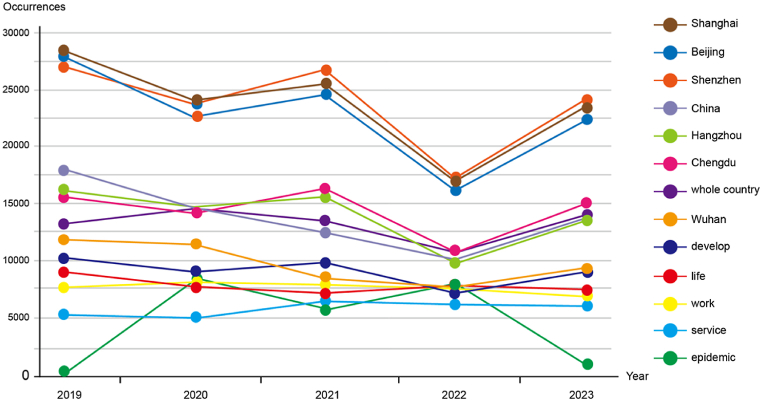
Trends of keywords occurring in all five years.

Fig. 1 shows that through the TF-IDF analysis of the text data of Weibo in Guangzhou from 2019 to 2023, the image perception trend of Guangzhou city is deeply explored. The data takes the year as the timeline, and keywords such as Shanghai, China, China, Beijing, development, work, Chengdu, service, Hangzhou, Wuhan, Shenzhen, life and epidemic situation as the vertical axis. Each data point represents the TF-IDF value of the keyword in the corresponding year. During the five-year study, the TF-IDF value of "Shanghai" increased from 28,330 in 2019 to 24,080 in 2023, reflecting the dynamic changes in the relationship and influence between Guangzhou and Shanghai. Keywords such as "development", "work" and "service" also showed a unique fluctuation trend in this period, which revealed the special development track of various fields of Guangzhou city and provided clues for further understanding the perception of Guangzhou city in economy, employment and service. At the same time, the TF-IDF values of "life" and "epidemic situation" experienced ups and downs in five years, reflecting the dynamic evolution of social life and public health, reflecting the sensitivity of urban residents to the quality of life and the focus of public attention during the epidemic period. This quantitative analysis provides a unique perspective for the perception of Guangzhou's city image, which enables us to understand the importance and evolution trend of keywords inside and outside the city more comprehensively.

### 4.4 Topic mining

**Fig. 2:**
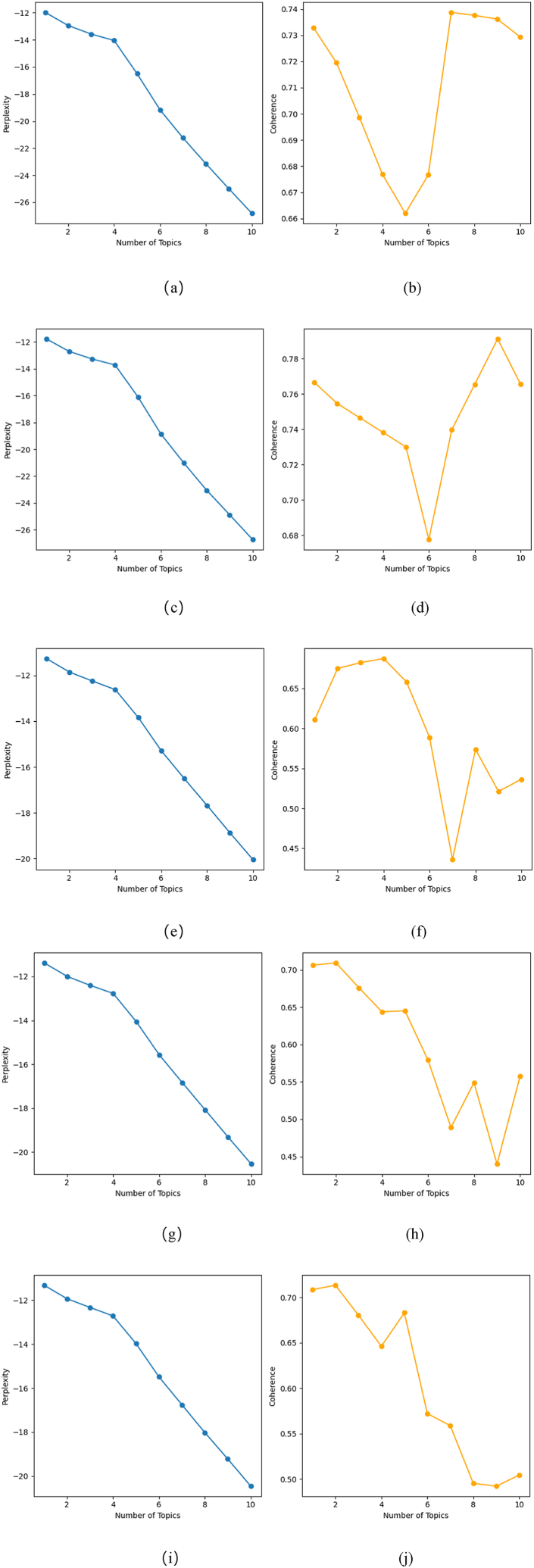
(a) Perplexity of the 2019 LDA topic model; (b) coherence of the 2019 LDA topic model; (c) perplexity of the 2020 LDA topic model; (d) coherence of the 2020 LDA topic model; (e) perplexity of the 2021 LDA topic model; (f) coherence of the 2021 LDA topic model; (g) perplexity of the 2022 LDA topic model; (h) coherence of the 2022 LDA topic model; (i) perplexity of the 2023 LDA topic model; (j) coherence of the 2023 LDA topic model.

Fig. 2 illustrates the consistency and confusion of the LDA theme model across various topics from 2019 to 2023. A line chart is created using Matplotlib in Python to determine the optimal number of topics, depicting the trade-off between consistency and confusion. Following the principle of high consistency and low confusion, this study identifies the suitable range for the number of topics each year as [1,10]. The curve exhibits a noticeable inflexion point at K = 4, indicating optimal performance. Therefore, this study sets the number of LDA topics to 4, ensuring the rationality and effectiveness of the model.

The LDA theme model analysis for 2019 (Table 7) reveals distinct user concerns on Weibo. In Theme 1, keywords like 'life,' 'like,' 'video,' and 'many' highlight the diversity of daily life, preferences, and cultural experiences users share, emphasizing Weibo's role in connecting users through shared interests. Theme 2 focuses on city names like 'Shanghai,' 'Beijing,' 'Chengdu,' and 'Hangzhou,' reflecting the collaborative dynamics between cities. Theme 3 showcases user interest in 'development,' 'China,' 'construction,' and 'service,' underlining the platform's significance in national, city, and enterprise development discussions. Keywords related to construction projects underscore users' deep concern for economic development and urban planning. In Theme 4, keywords such as 'house price,' 'Beijing,' 'Shenzhen,' and 'first-tier' highlight user concerns about first-tier cities' development, the real estate market, and economic trends. The discussion focuses on housing prices, and economic data reflects users' sensitivity to economic situations and real estate market dynamics.

**Table 7:** LDA topic model results of 2019.

Topic1	Probability Distribution	Topic2	Probability Distribution	Topic3	Probability Distribution	Topic4	Probability Distribution
Words		Words		Words		Words	
life	0.005	Shanghai	0.024	develop	0.009	house price	0.017
like	0.004	Beijing	0.023	China	0.007	Beijing	0.012
video	0.004	Chengdu	0.019	build	0.005	Shenzhen	0.012
many	0.004	China	0.017	service	0.004	first-tier	0.012
real	0.004	Hangzhou	0.017	enterprise	0.004	market	0.009
Weibo	0.003	Shenzhen	0.016	project	0.003	rise	0.008
time	0.003	Wuhan	0.015	center	0.003	Shanghai	0.008
delicacy	0.003	2019	0.011	2019	0.003	IELTS	0.008
interlinkage	0.003	Chongqing	0.011	World club cup	0.003	2019	0.007
China	0.003	Xi'an	0.011	5G	0.003	Ring ratio	0.006
webpage	0.003	Tianjin	0.010	Guangzhou City	0.003	price	0.005
place	0.003	Nanjing	0.010	The Greater Bay Area	0.003	real-estate market	0.005
Shenzhen	0.003	whole country	0.008	whole country	0.003	fall	0.005
work	0.002	Zhengzhou	0.008	work	0.003	housing	0.005
friends	0.002	Changsha	0.007	company	0.003	descend	0.005

The 2020 LDA theme model analysis (Table 8) unveils Weibo users' predominant concerns across various themes. In Theme 1, keywords like 'development,' 'China,' 'construction,' and 'project' underscore robust user interest in national, city, and enterprise development, particularly in construction projects and industrial planning. Theme 2 features keywords such as 'life,' 'video,' 'Weibo,' and 'many,' showcasing users' diverse content sharing on Weibo, emphasizing the platform's role in exchanging life trivia, preferences, and cultural experiences. Theme 3 centers around city names like 'Beijing,' 'Xi'an,' 'Chengdu,' and 'Shanghai,' coupled with travel-related words, reflecting user discussions on urban dynamics, travel planning, and transportation modes. Lastly, Theme 4 highlights keywords like 'Shenzhen,' 'Beijing,' 'Shanghai,' and 'house price,' indicating user concerns about first-tier city development, the real estate market, and economic trends. Housing prices and economic data take center stage in user discussions, revealing heightened sensitivity to economic situations and real estate market dynamics.

**Table 8:** LDA topic model results of 2020.

Topic1	Probability Distribution	Topic2	Probability Distribution	Topic3	Probability Distribution	Topic4	Probability Distribution
Words		Words		Words		Words	
develop	0.010	life	0.005	Beijing	0.011	Shenzhen	0.021
China	0.007	video	0.005	Xi'an	0.010	Beijing	0.017
build	0.006	Weibo	0.004	Chengdu	0.010	Shanghai	0.015
project	0.005	many	0.004	Shanghai	0.009	Hangzhou	0.012
enterprise	0.005	like	0.003	Chongqing	0.009	2020	0.010
work	0.004	real	0.003	whole country	0.009	China	0.010
service	0.004	time	0.003	airport	0.008	house price	0.009
country	0.004	library	0.003	Gansu province	0.007	Chengdu	0.009
Guangzhou City	0.004	Shanghai	0.003	Jiangsu Province	0.007	whole country	0.009
center	0.004	China	0.002	Wuhan	0.007	first-class	0.008
international	0.004	Shenzhen	0.002	interlinkage	0.007	Chongqing	0.007
2020	0.003	Beijing	0.002	Zhang yaonian	0.007	Nanjing	0.007
company	0.003	place	0.002	webpage	0.007	rise	0.007
whole country	0.003	work	0.002	set on a journey	0.007	data	0.007
industry	0.003	culture	0.002	Changsha	0.006	first	0.006

The LDA theme model analysis for 2021 (Table 9) reveals diverse concerns among Weibo users across four themes. In Theme 1, keywords like 'culture,' 'brand,' and 'activity' indicate high user interest in cultural promotion and brand activities, particularly those in 2021, highlighting a focus on cultural events. Theme 2 showcases active discussions on orthodontics topics, featuring keywords such as 'teeth,' 'orthodontics,' 'hospitals,' and 'doctors,' emphasizing users' deep concern for oral health and medical services, specifically in orthodontics. Theme 3 captures users sharing daily life trivialities and expressing emotions through keywords like 'life,' 'work,' and 'housekeeping,' emphasizing the themes of everyday matters and emotional expression. Lastly, in Theme 4, users exhibit keen interest in urban development and the service industry, with keywords like 'city,' 'development,' 'service,' and 'logistics,' mainly focusing on the development trends of cities like Shenzhen and Beijing, highlighting themes of urban development and the service industry.

**Table 9:** LDA topic model results of 2021.

Topic1	Probability Distribution	Topic2	Probability Distribution	Topic3	Probability Distribution	Topic4	Probability Distribution
Words		Words		Words		Words	
China	0.009	tooth	0.034	life	0.005	Shenzhen	0.008
culture	0.006	correct	0.031	work	0.005	develop	0.008
interlinkage	0.006	hospital	0.018	household management	0.004	Beijing	0.006
brand	0.006	doctor	0.016	many	0.004	Shanghai	0.006
2021	0.006	Beijing	0.014	China	0.003	whole country	0.006
webpage	0.006	Shanghai	0.014	real	0.003	China	0.005
activity	0.005	Chengdu	0.013	place	0.003	Foshan	0.005
Shanghai	0.005	Shenzhen	0.013	time	0.003	build	0.004
Chengdu	0.004	case	0.011	like	0.003	service	0.004
video	0.004	Hangzhou	0.011	video	0.002	Chengdu	0.004
travel	0.004	share	0.010	friends	0.002	2021	0.004
Beijing	0.004	oral cavity	0.009	hope	0.002	enterprise	0.004
Weibo	0.003	braces	0.008	epidemic	0.002	logistics	0.004
Guangdong	0.003	orthodontics	0.008	Weibo	0.002	Hangzhou	0.003
design	0.003	information	0.007	feel	0.002	epidemic	0.003

In the 2022 LDA theme model analysis (Table 10), Theme 1 emphasizes user concerns about epidemic prevention and control, service provision, and related measures, reflecting a continued focus on health and life safety. Theme 2, with keywords like 'housekeeping,' 'tourism,' 'video,' and 'Weibo,' indicates user interests in housekeeping services, travel experiences, and social media platforms, expanding beyond health into life entertainment and social interaction. Theme 3 explores users' discussions on life trivialities, work situations, and life in big cities through keywords like 'many,' 'life,' 'work,' and 'big city,' showcasing the diverse content shared on Weibo. Lastly, in Theme 4, keywords such as 'development,' 'China,' 'economy,' and 'enterprise' reveal user concerns for the country, city, and economic development, suggesting a focus on social progress, business trends, and future development directions.

**Table 10:** LDA topic model results of 2022.

Topic1	Probability Distribution	Topic2	Probability Distribution	Topic3	Probability Distribution	Topic4	Probability Distribution
Words		Words		Words		Words	
epidemic	0.023	household management	0.007	many	0.006	develop	0.007
service	0.021	China	0.006	life	0.005	China	0.007
prevention and control	0.014	travel	0.005	real	0.005	whole country	0.006
nucleic acid	0.013	video	0.005	work	0.004	Shenzhen	0.006
hour	0.011	Shanghai	0.005	like	0.004	Shanghai	0.006
personnel	0.009	Beijing	0.005	place	0.004	Beijing	0.006
provide	0.008	interlinkage	0.005	epidemic	0.004	Chengdu	0.004
examine	0.008	webpage	0.005	hope	0.003	build	0.004
life	0.007	2022	0.005	time	0.003	economy	0.004
health	0.007	Weibo	0.005	Shenzhen	0.003	2022	0.004
measure	0.007	Chengdu	0.004	friends	0.003	data	0.004
Infected person	0.007	subway	0.004	big city	0.003	market	0.004
product	0.006	Shenzhen	0.004	feel	0.003	enterprise	0.004
hotline	0.005	airport	0.004	Shanghai	0.002	project	0.003
zone	0.005	set on a journey	0.004	let go	0.002	product	0.003

The 2023 LDA theme model analysis (Table 11) reveals distinct user concerns across four themes. In Theme 1, keywords like 'Shanghai,' 'Beijing,' and 'Shenzhen' underscore users' heightened attention to these cities, with mentions of 'May Day Holiday' and 'Tourism' suggesting an interest in holiday travel and tourism activities. Theme 2 highlights 'development' and 'China' as primary keywords, indicating user concerns about national development and economic cooperation, with 'Greater Bay Area' and 'culture' hinting at interest in Greater Bay Area construction and cultural innovation. Theme 3 showcases keywords like 'subway,' 'market,' and 'studying abroad,' reflecting user concerns about urban transportation, market conditions, and information related to studying abroad. Lastly, in Theme 4, 'housekeeping' and 'life' dominate, signaling user interest in housekeeping services and daily life trivia. The presence of 'Weibo' and 'Time' suggests a trend of users sharing life experiences and reflecting on time on the Weibo platform.

**Table 11:** LDA topic model results of 2023**.**

Topic1	Probability Distribution	Topic2	Probability Distribution	Topic3	Probability Distribution	Topic4	Probability Distribution
Words		Words		Words		Words	
Shanghai	0.017	Development	0.014	Subway	0.010	household management	0.007
Beijing	0.015	China	0.010	market	0.006	life	0.005
Shenzhen	0.014	build	0.006	company	0.005	real	0.005
Chengdu	0.012	The Greater Bay Area	0.005	Study Abroad	0.005	Shanghai	0.004
Hangzhou	0.011	culture	0.005	project	0.005	mant	0.004
Video	0.009	Guangdong	0.004	Operation	0.005	Shenzhen	0.004
whole country	0.009	activity	0.004	service	0.004	time	0.004
Growth	0.008	industry	0.004	car	0.004	Like	0.004
Chongqing	0.008	Guangzhou	0.004	Policy	0.004	Video	0.003
Data	0.008	Center	0.004	Shenzhen	0.004	Beijing	0.003
May Day holiday	0.007	Economy	0.004	Shanghai	0.004	feel	0.003
Tourism	0.007	innovation	0.004	high-speed rail	0.003	place	0.003
Show	0.007	country	0.004	applying for	0.003	work	0.003
China	0.007	high quality	0.004	enterprise	0.003	Weibo	0.003
Wuhan	0.007	Cooperation	0.004	Investment	0.003	friend	0.002

### 4.5 Sentiment analysis

**Fig. 3:**
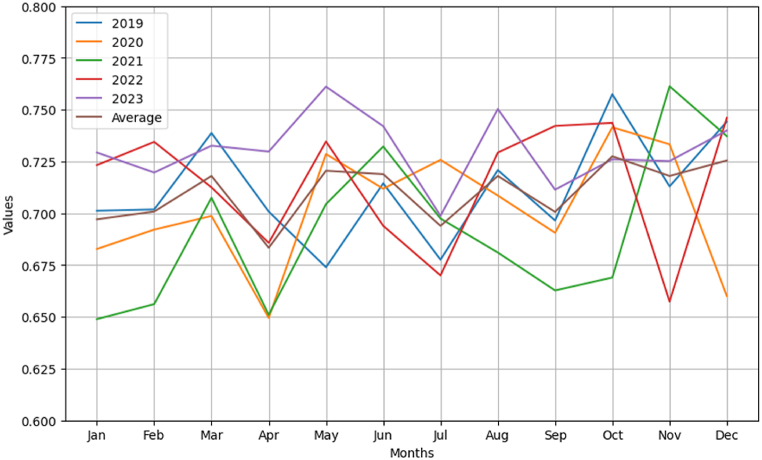
Trends in sentiment value from January to December 2019–2023.

Through SnowNLP emotional analysis, Guangzhou's emotional values over January to December 2019–2023 are quantified and categorised into three ranges: 0–0.4 for negative, 0.4–0.6 for neutral, and 0.6–1 for positive. Fig. 3 depicts the trend of emotional values from January to December 2019–2023. In the past five years, the emotional analysis of Guangzhou cities showed a positive trend. This can reflect the positive development of Guangzhou's overall social and economic environment. During this period, the positive emotional value is dominant, indicating that the market is optimistic about the development of Guangzhou. In 2020, although the world is facing uncertainties and challenges, Guangzhou's emotional analysis is still positive, which may reflect the city's resilience and adaptability in coping with challenges. The fluctuation of neutral emotional value may reflect the uncertainty of the market for some changes.

However, the neutral emotional value is relatively high overall, indicating that Guangzhou has maintained a relatively stable social environment in the past five years. The negative emotional value is relatively low, indicating that the market in Guangzhou has not suffered a significant negative impact during this period. This may be related to the city's economic growth, social stability and effective government management. It should be noted that sentiment analysis results may be influenced by factors such as data source and the choice of sentiment value range.

**Fig. 4:**
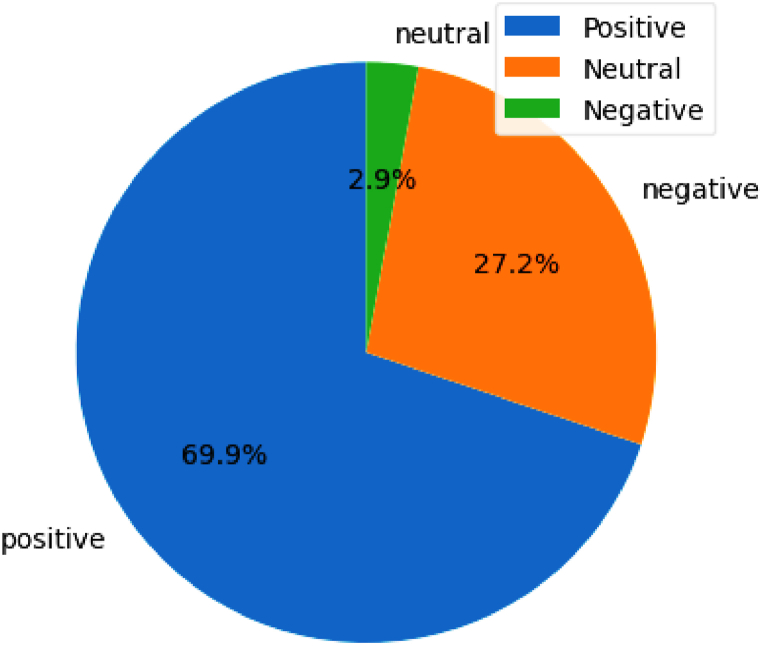
Distribution of sentiment values from 2019 to 2023.

In the past five years, the emotional analysis data of Guangzhou showed a positive trend in the city's image. According to the 377,430 pieces of Weibo data in Fig. 4, the average of all emotional values is 0.710, and the standard deviation is 0.415, of which the number of positive images accounts for 69.9 % of the total, of which 263,705 are positive, with an average of 0.974 and a variance of 0.005. This large number of positive comments reflects Guangzhou's economic, social and cultural prosperity, and citizens' confidence in the future of the city is very firm.

The number of neutral evaluations is 10,935, accounting for 2.9 % of the total, with an average of 0.502 and a variance of 0.003. This may reflect some fluctuations and uncertainties in the market. Although neutral evaluation is relatively rare, it reflects the market's concern about changes and challenges to some extent.

Finally, negative images accounted for 27.2 %, and there were 102,790 negative comments, with an average of 0.562 and a variance of 0.010. Although the quantity is relatively large, it is still a relatively small part compared with the total evaluation quantity. This may indicate that the negative impact on the market is relatively limited, and the city has dealt with potential difficulties relatively steadily in the past five years. Taken together, these data depict the optimistic picture of Guangzhou in the past five years, highlighting its strong economic, social and cultural performance. This has laid a solid foundation for the city's sustainable development in the future and also reminds us to continue to pay attention to the changes in the market to better adapt to future challenges.

## 5 Further discussion

### 5.1 Evolution of user interest in weibo

#### 5.1.1 2019–2020: science and technology and epidemic situation play a dual leading role

In 2019, Guangzhou became the core city of the Greater Bay Area, attracting the attention of a wide range of Weibo users. Science and technology and regional development have become the leading keywords, reflecting users' strong interest in scientific and technological progress and regional development. The keywords "5G″ and The Greater Bay Area" highlight users' strong concern about scientific and technological progress and regional development. The promotion of the Greater Bay Area Plan and the gradual landing of 5G technology have become hot topics, reflecting Guangzhou's position as a scientific and technological innovation and economic center. However, the outbreak of the COVID-19 pandemic in 2020 completely changed the interest focus of users on Weibo. The epidemic situation has become the dominant topic. Guangzhou, as the key node of epidemic prevention and control, has aroused users' continuous attention to keywords such as "nucleic acid detection" and "prevention and control", highlighting the urgent demand of Guangzhou residents for epidemic information. The changes in this period reflected the sensitivity of users' focus on Weibo, which changed from the rapid development of science and technology and regions to great concern for public health and epidemic prevention measures.

#### 5.1.2 2021–2023: epidemic evolution and multiple concerns

Over time, users on Weibo gradually freed themselves from the concern of the epidemic and began diversifying their interests. The emergence of new keywords such as "doctor" and "enterprise" shows the change in users' interests. During this period, the rise of the keywords "doctor" and "enterprise" may reflect the social adjustment and economic recovery after the epidemic. Guangzhou has become one of the economic centers of gravity, and the attention of enterprises and medical resources has increased. At the same time, the impact of real-time events in Guangzhou is also deeply reflected in the concerns of users on Weibo, including urban planning, infrastructure construction, economic activities and other initiatives. Continued attention to keywords such as "house price", "enterprise" and "economy" may be closely related to real-time events such as Guangzhou's economic development and real estate market fluctuation. In addition, the emergence of keywords such as "oral health" and "studying abroad" reflects the increasing concern of Guangzhou residents in the fields of health and education. By combining the real-time events in Guangzhou with the evolution of users' interests in Weibo, we have a more comprehensive understanding of the dynamic changes in public opinion in Weibo during this period, as well as users' concerns about urban development and public health.

### 5.2 Characterization of Guangzhou's city image

Guangzhou has rich historical and cultural resources, as an important international trade port and exchange center, Guangzhou's international image is also very prominent on Weibo. LDA analysis digs deeper into specific topics and reveals users' interests in different areas. Keyword themes such as “life”, “development”, and “house price” represent the various aspects of Guangzhou's discussion among Weibo users. Secondly, the theme of “life” may reflect concerns about the quality of life of urban residents and the social environment. Meanwhile, the theme of “development” highlights users' expectations of Guangzhou's economic, technological and cultural progress. Second, city-related keywords such as “Beijing,” “Shanghai,” and “Guangdong” appear frequently on Weibo, indicating users' continued interest in different cities in China. This suggests that users are engaged in the dynamics of competition and cooperation between cities and their respective trajectories. Third, Guangzhou's international image as a major international trade port and exchange center is also prominent on Weibo. Posts about the Canton Fair, international exchange events and multicultural communities demonstrate Guangzhou's openness and tolerance as a cosmopolitan city. Fourth, keywords such as “enterprise” and “economy” show that people are increasingly interested in Guangzhou's economy and business trends, influenced by the city's status as an economic and innovation center. Users' interest in business development and economic patterns reflects Guangzhou's image as a prosperous city.TF-IDF and LDA analyses quantify user engagement and reveal the multifaceted nature of microblogging perceptions of the city, providing insights into Guangzhou's image on social media.

Finally, the article also points out that Guangzhou's city image is susceptible to national events, especially controversial ones, which may change the emotional inclination of some people around the world towards Guangzhou in a short period of time.

### 5.3 Communication strategy for Guangzhou's urban image

City image communication is an important part of modern city development strategy, which is crucial for enhancing the city's popularity, attractiveness and competitiveness. As the economic center of southern China and an important international metropolis, Guangzhou's urban image communication strategy needs to consider various aspects such as culture, economy, science and technology, and livability. The following is the communication strategy of Guangzhou's urban image.

1. Combination of multiculturalism and modernized city image. Guangzhou has a rich cultural heritage and modern urban construction, and the combination of the two is key to shaping its unique urban image. Among the keyword adjectives, words such as “culture” and “activities”, which are always associated with the city, are often associated with positive content, giving people a positive impression of Guangzhou. Literature shows that city branding can enhance the attractiveness of a city by integrating traditional culture and modern elements [[43], [44], [45]]. Guangzhou has gained widespread attention for its technological advances and regional development during 2019–2020, as indicated by Table 2. By continuing to publicize Guangzhou's achievements in 5G technology and the construction of the Greater Bay Area, it can further consolidate its image as a center of science, technology, innovation and economy. Promote Guangzhou's achievements in high-tech and innovative industries and organize technology expos and innovation and entrepreneurship forums to attract global technology companies and talents.
2. The development of cultural tourism routes is an essential way to enhance the image and attraction of tourist cities. The research shows that cultural tourism has a significant impact on the city image and can promote the sustainable development of the city under the pressure of tourism [46]. Designing tourism routes covering Lingnan architecture, folk culture, and traditional handicrafts to attract tourists to experience the cultural charm of Guangzhou. Plan tourism routes themed on authentic Guangzhou cuisine to attract domestic and foreign tourists through food.
3. Media cooperation and publicity, the media plays an important role in the communication of city image. Cooperation with domestic and international mainstream media can effectively enhance the city's international popularity and reputation [47,48]. Produce special programs showcasing Guangzhou's achievements in culture, economy, science and technology to attract a global audience. Write and publish in-depth reports and articles to deeply analyze Guangzhou's unique charm and development potential. Collaborate with mainstream media to publish news reports on Guangzhou's development to enhance the city's exposure.
4. Big data monitoring and feedback. The application of big data and artificial intelligence technology in city image communication is becoming more and more widespread, which can help cities monitor and analyze public feedback on city image in real time [28,29,31]. Big data platforms are used to monitor city image feedback on social media such as microblogging and Tiktok platform to analyze public opinion. Through sentiment analysis tools, understand the public's emotional tendency towards the city image and adjust the propaganda content in time. Regularly evaluate the effect of communication strategies, optimize the publicity content and channels, and improve the efficiency of communication.
5. Enhance the construction of community and public space. Enhancing the construction of community and public space can enhance the sense of belonging of citizens and the experience of tourists, thus improving the overall image of the city [49]. Improve the construction of urban public facilities to enhance the convenience and comfort of citizens and tourists. Organize community cultural activities to enhance citizens' sense of participation and improve the image of the city. Increase urban greening efforts to create a green and livable environment and improve the overall image of the city.

Through the above strategies, Guangzhou can further spread its city image, stimulate tourism vitality, enhance the city's tourism competitiveness, and realize the city's sustainable development. These strategic measures can not only help Guangzhou enhance its international image, but also strengthen the city's comprehensive competitiveness and lay a solid foundation for its future development.

### 5.4 Discussion

In this paper, we searched the Web of Science (https://www.webofscience.com/wos/woscc/basic-search, accessed on January 10, 2023) for papers on the topic of “urban image”. Through browsing and organizing, we found that most of the research on urban image are qualitative and lack sufficient data support. Subsequently, we set the topics as “urban image”, “Guangzhou” and “microblogging”, and found that there were no papers related to this topic. Later, we adjusted the theme to “city image” and “Guangzhou”, and found that there were four related papers. Among them, one paper studied the perception of Guangzhou's urban image by different generations, and explored the similarities and differences in the perception of urban image among different age groups [50]. Another paper analyzed the construction of city image in Guangzhou metro station advertisements in the context of globalization, exploring how advertisements use globalization elements to shape and spread Guangzhou's city image, reflecting the city's self-positioning and publicity strategies in the process of globalization [45]. The third paper examines the relationship between immigration, race and city image, especially how Guangzhou manages and presents its city image by emphasizing “cleanliness, safety and orderliness” in the context of globalization [51]. The final article explores the expression and transmission of Guangzhou's urban image in the historical period by analyzing the Pearl River barge boards and the Qing Dynasty Guangzhou cityscape maps. The study focuses on the city image and its evolution as reflected in historical relics and documents [52].

To summarize, there is a lack of case studies on urban image research; it is noteworthy that there are few studies on Guangzhou's image. Most of these analyses are qualitative, and there are fewer studies on city image based on social media platform using text analysis technology. This paper further expands the research in related fields and enriches the research results. It has also made contributions in methodology, theory and practical application, mainly in the following three aspects.

1. From a methodological point of view, the study further explores Guangzhou's urban image based on the data of tweet text, keyword extraction by TF- IDF method, theme analysis by LDA model, and sentiment analysis by SnowNLP method. The combination of these three methods can more comprehensively analyze users' perceptions of Guangzhou's urban image. A social media platform text mining framework for city image research is formed.
2. From the perspective of theoretical application, this study enriches the theory of urban image, especially in the context of the digital age and social media. By combining traditional urban image research methods with modern natural language processing techniques, this study provides a new theoretical perspective that explains the importance and application potential of social media data in urban image research. This not only extends the existing theoretical framework, but also provides new paths and methods for future research.
3. In terms of practical application, the study provides valuable reference data for city managers and decision makers. By analyzing the discussions and emotional tendencies of Weibo users towards Guangzhou, the study reveals the public's views and emotions towards different aspects of the city. This information can help policy makers identify strengths and weaknesses in the city's image, so that they can formulate targeted improvement measures to enhance Guangzhou's overall image and attractiveness. At the same time, the methodology and results of this study provide a framework and practical experience that can be utilized by other cities to conduct similar urban image studies.

## 6 Conclusion

In this paper, we use web crawler technology to collect tweets about “Guangzhou” in Weibo from 2019 to 2023, and analyze the city image of Guangzhou through TF-IDF, LDA and SnowNLP sentiment analysis. On the basis of the analysis results, strategies to improve the competitiveness of urban tourism are proposed. According to the research analysis, Guangzhou's city image on Weibo presents the following characteristics:(1) As the economic center of southern China, Guangzhou often displays its prosperous business environment and innovative technology industry. Many Weibo users share information about business opportunities, investment environment and high-tech enterprises in Guangzhou. (2) As an important international trade port and exchange center, Guangzhou's international image is also prominent on Weibo. Posts about the Canton Fair, international exchange activities and multicultural communities demonstrate Guangzhou's openness and tolerance as a cosmopolitan city. (3) Guangzhou has rich historical and cultural resources, such as Lingnan culture, Cantonese opera and cuisine. Discussions and sharing of Guangzhou's cultural activities, historical relics, traditional festivals and specialty cuisines are often found on Weibo, demonstrating Guangzhou's deep cultural heritage and unique regional flavor. (4) Guangzhou's urban planning, greening and convenience of life are also well received on Weibo. Many residents and visitors would share Guangzhou's parks, leisure facilities and modern living conditions, highlighting its livability.

Through the analysis of Guangzhou's urban image and its presenting characteristics, the strategy of Guangzhou's urban image is further proposed from the perspective of communication, including the following steps: (1) Based on the characteristics of Guangzhou's multiculturalism, modernized metropolis, economic vitality and livability, the city positioning of Guangzhou is clarified, and it creates an “international metropolis where culture and modernity coexist” brand. (2) Develop special cultural tourism routes in Guangzhou, such as “Lingnan Culture Exploration” and “Gourmet Tour”, to enhance the experience of tourists. (4) Establish long-term partnerships with domestic and international mainstream media, and continue to disseminate Guangzhou's city image through news reports, special programs and in-depth articles. (5) Utilize big data and artificial intelligence technology to monitor real-time feedback on the city's image in social media and news reports, and adjust communication strategies in a timely manner.

The main implications of this study are as follows. (1) Quantifying keyword importance, identifying potential themes, and revealing sentiment tendencies by integrating TF-IDF, LDA, and sentiment analysis. (2) Using Guangzhou as an example, it provides valuable insights for policy makers and urban planners to identify key areas and sentiment tendencies to guide improvement and strategic initiatives to enhance the city's image and sustainable development. (3) Demonstrate the potential of digital tools and social media data, emphasize the effectiveness of online platforms and natural language processing techniques in large-scale, real-time urban image analysis, and provide new perspectives for other cities.

However, this study currently has some limitations, and future research includes (1) The data of the study mainly comes from the microblogging platform, which may have a certain sample bias and cannot fully represent the voices of all residents in Guangzhou, and future research can combine the data from other social media platforms, such as Little Red Book and Tiktok platform, etc., in order to obtain a more comprehensive public opinion. (2) Despite the use of TF-IDF, LDA and sentiment analysis methods, these analysis tools have their limitations and cannot cover the complex emotions and opinions of all microblogging users. (3) This paper mainly uses text data, and in the future, data types can be further expanded to include images, videos, audio, etc., to study city image from multiple perspectives. For example, image recognition technology can help analyze the visual image of the city, while video and audio analysis can capture public feedback in terms of dynamics and sound. (4) Future research can compare and analyze Guangzhou with other cities to understand the differences and commonalities of urban image in different cultural contexts and provide a broader reference. (5) Through time series analysis, a longer time span study can be conducted to observe the trend of city image change over time and identify the long-term factors affecting the city's image. (6) Combine quantitative analysis with qualitative research, and through field surveys and interviews, gain a deeper understanding of the public's true feelings about city image to make up for the shortcomings of big data analysis.

## Funding statement

This research did not receive any specific grant from funding agencies in the public, commercial, or not-for-

profit sectors.

## Data availability

Data will be made available on request.

## CRediT authorship contribution statement

**Huimin Qu:** Writing – original draft, Visualization, Software, Resources, Methodology, Investigation, Formal analysis, Conceptualization. **Bor Tsong Teh:** Writing – review & editing, Supervision, Methodology. **Nikmatul Adha Nordin:** Supervision, Methodology. **Zhuqin Liang:** Investigation.

## Declaration of competing interest

The authors declare that they have no known competing financial interests or personal relationships that could have appeared to influence the work reported in this paper.
